# Vowel space change as a predictor of speech intelligibility gain in Down syndrome

**DOI:** 10.1080/02699206.2026.2613817

**Published:** 2026-01-22

**Authors:** Delin Deng, Stephen Camarata, Julie Mazzone, Miriam Lense, Duane Watson

**Affiliations:** a Department of Psychology and Human Development, Vanderbilt University, Nashville, Tennessee, USA;; b Department of Hearing and Speech Sciences, Vanderbilt University Medical Center, Nashville, Tennessee, USA;; c Department of Otolaryngology, Vanderbilt University Medical Center, Nashville, Tennessee, USA

**Keywords:** Vowel space, Down syndrome, children, intelligibility

## Abstract

Reduced intelligibility is a frequent characteristic of the speech of children with Down syndrome (DS). While it is documented that compressed vowel space is often associated with low intelligibility, few studies have examined how acoustic vowel space changes during speech enrichment interventions are related to functional intelligibility gains. This study investigated whether change in acoustic vowel space can predict intelligibility improvement in DS. Thirteen children with DS completed a naturalistic speech enrichment intervention and were included in the present analysis. Vowel productions from a structured /hVt/ word task were acoustically analysed at baseline and post-speech enrichment interventions. To measure vowel space change, three metrics were used: articulatory-acoustic vowel space (AAVS), quadrilateral vowel space area (qVSA), and convex hull vowel space area (VSAhull). Intelligibility gains were measured using Growth Scale Values (GSVs) from the Woodcock Camarata Articulation Battery (WCAB). Regression analysis results revealed that changes in AAVS and VSAhull significantly predicted WCAB gains, while qVSA did not. These findings suggest that vowel space change reflects clinically meaningful articulatory adjustments, as indicated by their predictive association with intelligibility improvement following speech enrichment interventions. The lack of predictive power for qVSA highlights limitations of traditional corner-vowel metrics and supports the use of more comprehensive vowel space measures. While preliminary, these findings suggest that vowel space metrics may have future potential for clinical monitoring of speech development in children with DS, pending further validation in larger and more controlled studies.

## Introduction

Speech intelligibility, defined as ‘how well a speaker’s acoustic signal can be accurately recovered by a listener’ (Hustad, 2008: 562), is a persistent and well-documented challenge for individuals with Down syndrome (DS). This reduced intelligibility has significant social and functional consequences, limiting everyday communication and participation (Barnes et al., 2009; Bray & Woolnough, 1988; Bunton et al., 2007; Kumin, 1994, 2006; Rosin et al., 1988). The underlying causes are multifactorial and include motor speech impairments, atypical prosody, and reduced articulatory precision (Carl et al., 2020; Cleland et al., 2010; Kenta & Vorperiana, 2013; Wild et al., 2018), although the relative contributions of each remain unclear.

Among the many approaches to evaluating speech production, articulatory vowel space area has emerged as a valuable acoustic metric for capturing articulatory precision. Vowel space is typically characterised by first and second formant frequencies (F1 and F2), which correspond to tongue height and tongue advancement, respectively. In typical speech, larger and more dispersed vowel spaces indicate clearer articulatory targets and have been associated with higher intelligibility (Bradlow et al., 1996; Prosek et al., 1987; Turner et al., 1995). Compressed or centralised vowel spaces are frequently observed in disordered speech and are associated with reduced intelligibility. This pattern has been documented in motor speech disorders such as dysarthria and apraxia of speech, as well as in speech delays associated with developmental conditions (Bunton, 2006; Higgins & Hodge, 2002; Zajac et al., 2006).

Traditionally, vowel space has been measured using the quadrilateral vowel space area (qVSA), defined by the four corner vowels /i/, /u/, /æ/, and /ɑ/ (Vorperian et al., 2023). While qVSA provides a convenient summary of articulatory range in English, it may fail to capture non-uniform or participant-specific vowel distributions, especially in clinical populations (Bunton & Leddy, 2011) or in languages with fewer than four corner vowels, where triangular configurations are more appropriate. To address this, more comprehensive metrics have been proposed, such as convex hull vowel space area (VSAhull), which includes all vowel tokens within the smallest convex polygon (Luan et al., 2014; Story & Bunton, 2017), and articulatory-acoustic vowel space (AAVS), which quantifies the statistical dispersion of F1-F2 values across the vowel inventory (Palmer, 2015; Whitfield & Goberman, 2014).

These newer metrics provide a more nuanced and individualised view of articulatory behaviour and have demonstrated accuracy in capturing articulatory patterns in adult neurogenic populations. However, to our knowledge, no prior studies have applied AAVS or VSAhull to children with DS, nor have they evaluated their relationship to functional outcomes such as intelligibility.

In addition to qVSA, AAVS, and VSAhull, other vowel space metrics have been used to characterise articulatory precision, particularly in DS research. The Formant Centralization Ratio (FCR; Sapir et al., 2010) offers a measure of vowel centralisation by calculating the proximity of formants to the centre of the vowel space. The DS-VR ratio (F2 i/u; Moura et al., 2008) captures the contrast between front and back vowels and has been applied in studies of DS speakers (Carl & Icht, 2025). Another promising measure is vowel cluster size, which reflects within-category consistency and articulatory dispersion across repetitions (Carl & Icht, 2025; Carl et al., 2020). While these metrics were not used in the present study, they represent valuable alternatives for future work. Our study focused on AAVS, qVSA, and VSAhull due to their geometric interpretability and prior use in detecting articulatory change in adult speech populations. Their application to developmental speech disorders remains a promising direction for future research.

In children with DS, most intelligibility assessments rely on perceptual tools. Standardised measures such as the Percentage of Consonants Correct (PCC) (Barnes et al., 2009; Kennedy & Flynn, 2003; McCann & Wrench, 2007; Roberts et al., 2005), the less frequently used Percentage of Vowels Correct (PVC) (van Bysterveldt, 2009; van Bysterveldt et al., 2010), and subjective intelligibility ratings (Kumin, 2006) are commonly used to estimate speech accuracy. While perceptual ratings are sensitive to changes that affect functional intelligibility and may reflect articulatory variation indirectly, they do not provide detailed, quantifiable data on articulatory movement itself. In particular, acoustic measures can detect subtle changes in vowel production or motor coordination that may precede perceptual improvement or remain undetected by raters due to listener bias or variability. Additionally, these perceptual tools are often used at single time points rather than to track fine-grained articulatory progress across sessions (Darling-White & McHugh, 2025; O’Leary et al., 2020).

More recently, researchers have begun exploring acoustic vowel space metrics as dynamic indicators of articulatory development. In general, larger vowel spaces and reduced formant variability are associated with higher intelligibility across speech disorders (Carl et al., 2020; Sorianello, 2021; Thompson et al., 2023), though such findings have rarely been extended to intervention contexts in DS. One notable exception is the potential use of vowel space change to monitor intervention outcomes. Given that articulatory gains may precede perceptual intelligibility gains, especially in naturalistic or low-structure interventions, vowel space metrics offer a promising avenue for detecting sub-phonemic changes earlier in treatment progressions (Bunton & Leddy, 2011; Vorperian et al., 2023). Because of the wide heterogeneity in speech profiles among children with DS, group-level comparisons may obscure meaningful progress. Detailed, within-participant acoustic analyses provide a sensitive method for detecting articulatory changes that may not yet be reflected in perceptual scores, especially in early or naturalistic intervention contexts (Bunton & Leddy, 2011; Whitworth & Bray, 2015).

One assessment tool that is particularly well-suited to capturing such functional gains is the Woodcock Camarata Articulation Battery (WCAB) (Woodcock, 2019), which provides whole-word accuracy scores and computes Growth Scale Values (GSVs), norm-referenced scores that represent within-participant change across time. GSVs offer a clinically interpretable metric of speech intelligibility development that can complement acoustic data and reflect functional improvements resulting from intervention. Kwok et al. (2022) concluded that GSVs were not only psychometrically sound but also the most sensitive measure of direct changes in skills compared to raw, standard, and age-equivalent scores. Because most speech assessments do not include GSVs, the WCAB is especially useful for measuring change in speech ability.

Despite growing interest in acoustic predictors of speech intelligibility, few studies have examined how vowel space change during intervention relates to GSV-based intelligibility improvement in children with DS. The present study addresses this gap by analysing acoustic data from repeated /hVt/ word productions before and after a 3-month speech enrichment program. This approach, rooted in naturalistic interaction and broad target recasts, has been previously shown to support speech development in children with developmental speech disorders (Camarata et al., 2006; Yoder et al., 2005). However, it should be noted that the current study is not designed to test whether the treatment differentially improved speech. Rather than aiming to isolate direct treatment effects, the speech sessions in this study were designed to facilitate growth, with the goal of identifying predictors of intelligibility change regardless of source. Changes in three vowel space metrics (qVSA, VSAhull, and AAVS) were examined and their association with intelligibility gains, as measured by WCAB GSVs, was assessed.

The study addresses the following research questions:

1. Do children with DS demonstrate systematic changes in vowel space area following speech enrichment interventions?
2. To what extent are changes in acoustic vowel space measures (AAVS, qVSA, VSAhull) associated with gains in speech intelligibility, as indexed by GSV scores on the WCAB?

## Methodology

### Participants

Thirteen children with DS completed this study. Inclusion criteria were: (a) a confirmed diagnosis of DS, (b) age between 4 and 16 years, (c) use of 2–3 word utterances as determined by parent report and screening observations, (d) passing a hearing screening, and (e) English as the primary language spoken in the home. Exclusion criteria included the presence of uncontrolled seizures, diagnosed ADHD, apraxia secondary to a known neurological disorder, persistent stuttering, or severely disruptive behaviour as reported by parents. Written informed consent was obtained from all caregivers. Assent was also obtained from participants at the start of their first baseline session.

Table 1 presents detailed demographic information on these participants. As shown in Table 1, participants’ chronological ages ranged from 4 years, 2 months to 16 years, 3 months. Among these 13 children, there are 8 females and 5 males. Age-equivalent scores derived from the WCAB articulation test ranged from under 2 years, 0 month to 3 years, 3 months. These scores were used to characterise individual differences in speech intelligibility in speech intelligibility across participants.

### Study design and procedures

#### Speech and language assessment

To assess speech intelligibility, the WCAB was administered at both baseline and post-speech enrichment timepoints. The WCAB evaluates speech production accuracy at the whole-word level and provides GSVs, which reflect individual progress over time. Administration was conducted by a licenced speech-language pathologist (SLP) or by trained graduate or undergraduate research staff working under the direct supervision of an SLP. All assessors received formal training in WCAB administration and scoring procedures prior to data collection. During testing, the clinician presented each word aloud and the participant repeated it; responses were judged in real time as correct or incorrect based on whole-word accuracy. Although there are different methods for measuring intelligibility in the literature, the WCAB process is in accord with word level assessment associated with articulation testing (see, Stimley & Hambrecht, 1999). These binary judgements were guided by the WCAB scoring manual and recorded for each trial. To ensure standardisation and reduce scoring bias, fidelity monitoring was conducted through regular review of session recordings by a second trained rater on a subset of responses. GSVs were calculated from raw scores using standardised transformation procedures, and change in GSV from baseline to post-test served as the primary outcome measure of intelligibility improvement.

#### Acoustic vowel space task

Acoustic vowel space was examined to quantify articulatory function across a range of vowel categories. To elicit controlled productions, a clinician modelled 10 monosyllabic words designed to approximate an /hVt/ frame, with /h/ in initial position, a target vowel in medial position, and /t/ in final position when possible. The stimuli included: *heat, hit, het, hate, hat, hut, hoot, hood, hoed*, and *hot*. Most items were real English words; one non-word, *het*, was included to elicit the mid-front vowel /ɛ/, and *hoed* was used to elicit /o/, as no natural /hVt/ lexical items exist for these vowels. *Hood* was included to capture /ʊ/, serving as a near-minimal pair to *hoot* (/u/). These adaptations allowed for consistent coverage of 10 target vowels: /i/, /ɪ/, /ɛ/, /e/, /æ/, /ə/, /u/, /ʊ/, /o/, and /ɑ/. The central unrounded vowel corresponding to the STRUT lexical set (e.g. ‘hut’) was transcribed as /ə/ to maintain consistency and interpretability in the vowel space analyses.^1^

Each word was produced three times in immediate succession, in isolation (not within carrier phrases), following clinician modelling. Words were presented in a fixed order across participants. All productions were recorded using a Marantz PMD661 audio recorder and later analysed using Praat (Boersma & Weenink, 2025). While this approach ensured consistency, repeated sequential productions may have introduced practice or adaptation effects. Future studies may benefit from incorporating randomisation or interleaving to reduce potential confounds.

#### Speech enrichment interventions

These activities are referred to as speech enrichment interventions, acknowledging their purpose in supporting gains in speech intelligibility, despite the study not being structured to isolate treatment effects through a controlled design. Following baseline assessments, participants engaged in approximately 3 months of intervention focused on promoting phonological development and expressive communication. Interventions were scheduled once or twice weekly, lasting approximately 30 minutes per session. Depending on each participant’s needs and availability, sessions were conducted either in person or via a HIPAA-compliant telehealth platform. Across the program, participants received between 8 and 20 total sessions, with the number varying due to individual availability, illness, and scheduling constraints. Post-testing was conducted after the completion of the final intervention, not after individual sessions.

All speech enrichment interventions were naturalistic and play-based, designed to encourage spontaneous verbal interaction. Clinicians employed a Broad Target Speech Recast (BTSR) approach (Yoder et al., 2005), in which recasts were not restricted to developmentally appropriate targets but provided models of accurate adult speech across a broad phonemic inventory. For example, if a child said ‘wed’ for ‘red’, the clinician would respond with ‘red’, then pause before continuing the interaction. While /r/ was typically not expected to be mastered by all participants due to age, such forms were still recast as accurate models. Importantly, recasts included both consonantal and vocalic targets, offering consistent auditory models for vowels as well, particularly relevant to the vowel-based acoustic analyses in this study. These recasts were delivered at a rate of approximately three per minute and were intended to model accurate articulation, intonation, and stress without requiring imitation. While participants were not prompted to repeat the recasts, some spontaneously imitated the adult model.

To accommodate the broad age and developmental range of participants, clinicians individualised sessions by adjusting their interaction style, materials, and verbal expectations. For younger children or those with limited expressive language, sessions focused on simplified verbal routines, repetition, and lexical closure cues to support initiation. Older or more verbal children engaged in extended conversation, story-based play, or embedded learning tasks with richer phonological targets. In all cases, play materials and prompts were chosen to reflect the participant’s interests and developmental profile, ensuring the core recast strategy was accessible and appropriate across ages.

Although individual session data were not analysed for this study, the speech enrichment period provided the clinical context within which phonological and articulatory development occurred. Changes in vowel articulation and WCAB scores from baseline to post-speech enrichment interventions were used to assess the relationship between articulatory acoustic change and intelligibility gains.

#### Acoustic measurements and vowel space calculation

Trained research assistants manually segmented each vowel and measured the first (F1) and second (F2) formant frequencies at the temporal midpoint of the vowel. Formant values were extracted using a customised Praat script for each token, and average F1 and F2 values were computed per vowel per participant, separately for baseline and post-speech enrichment interventions.

Three vowel space metrics were derived from the F1 and F2 formant data:

Articulatory-Acoustic Vowel Space (AAVS) was calculated as the square root of the generalised variance of F1 and F2 values across all vowel tokens, providing a measure of overall articulatory variability (Whitfield & Goberman, 2014).

Quadrilateral Vowel Space Area (qVSA) was computed using the F1-F2 coordinates of the four corner vowels /i/, /u/, /æ/, and /ɑ/, following established protocols (e.g. Lansford & Liss, 2014; Tjaden et al., 2013; Turner et al., 1995; Whitfield & Goberman, 2017).

Convex Hull Vowel Space Area (VSAhull) was calculated using a convex hull algorithm to estimate the perimeter-based area enclosing all vowel tokens, thereby capturing maximum acoustic vowel space (e.g. Sandoval et al., 2013).

Each metric was calculated separately for baseline and post-speech enrichment interventions. Change scores (post minus baseline) were computed for use as predictor variables in subsequent statistical modelling.

#### Statistical analysis

To investigate whether changes in vowel articulation predicted gains in speech intelligibility, descriptive statistics were first examined for all phonemes (see Results). A multiple linear regression analysis was then conducted using R (R Core Team, 2025). The dependent variable was WCAB AE Gain, defined as the difference in GSV scores from the WCAB AE subtest between post-speech enrichment interventions and baseline sessions. Predictor variables included change scores for three acoustic vowel space metrics: Articulatory-Acoustic Vowel Space (AAVS), Quadrilateral Vowel Space Area (qVSA), and Convex Hull Vowel Space Area (VSAhull). The model was specified as follows:

lm(WCAB_AE_GAIN~ΔAAVS+ΔqVSA+ΔVSAhull,data=data)

All statistical modelling was conducted using the *lm()* function from the base stats package. Multicollinearity among predictors was assessed using Variance Inflation Factors (VIF) via the *vif()* function from the *car* package (Fox & Weisberg, 2019). Model summaries, coefficient estimates, and p-values were extracted using the *broom* package (Robinson et al., 2022) to ensure tidy and interpretable output. Diagnostic plots were visually inspected to verify assumptions of linearity, normality of residuals, and homoscedasticity.

This analysis was designed to evaluate the extent to which post-speech enrichment intervention changes in articulatory vowel space, as measured acoustically, explained individual differences in intelligibility gains.

## Results

### Descriptive statistics

Table 2 presents the mean first (F1) and second (F2) formant frequencies (in Hz) for each of the 10 target vowels produced by children with DS at the baseline session, alongside published reference values for typically developing (TD) children aged 10 to 12 years (Hillenbrand et al., 1995). This comparison allows for evaluation of group-level articulatory differences, particularly in tongue height and advancement, as indexed by F1 and F2, respectively.

Across both groups, high front vowels such as /i/ and /e/ were produced with low F1 and high F2 values, consistent with expected acoustic patterns. However, the DS group exhibited substantially lower F2 values across most vowels compared to their TD peers. For example, F2 for /i/ in the DS group averaged 1810 Hz, while TD children produced the same vowel at 3081 Hz. Similar F2 reductions were observed for /ɪ/ (1860 Hz in DS vs. 2552 Hz in TD), /e/ (1827 Hz vs. 2656 Hz), and /æ/ (1632 Hz vs. 2501 Hz), suggesting markedly less fronting of the tongue in the DS group. These reductions were not isolated to front vowels: even for back vowels such as /ɑ/, the DS group produced a lower F2 (1472 Hz) than TD children (1688 Hz), indicating overall reduced articulatory contrast along the front-back dimension.

Differences in F1 values were also observed, especially for low vowels. For /æ/, children with DS produced higher F1 values (903 Hz vs. 717 Hz in TD), indicating greater lowering relative to their TD peers. Conversely, for /ɑ/, the DS group showed lower F1 values (897 Hz vs. 1002 Hz in TD), which may reflect reduced articulatory displacement or variability in vowel target realisation. Some vowels, such as /ɛ/ and /u/, showed relatively small F1 differences between groups, while /ʊ/ had a noticeably higher F1 in the DS group (627 Hz vs. 568 Hz in TD), indicating less constriction for that vowel.

Overall, at baseline, the DS group’s vowel productions suggest a compressed and centralised vowel space, particularly along the F2 axis. This reduced acoustic distinction among vowels is consistent with prior findings of limited articulatory precision in individuals with DS and reinforces the need for acoustic measures that capture fine-grained articulatory variation when evaluating speech enrichment interventions outcomes.

To further illustrate the articulatory differences between children with DS and TD peers, a vowel space plot was generated using the average formant frequencies presented in Table 2. This visual comparison allows for a more intuitive examination of group-level contrasts in tongue height and advancement, as reflected in F1 and F2 acoustic dimensions.

**Figure 1.**
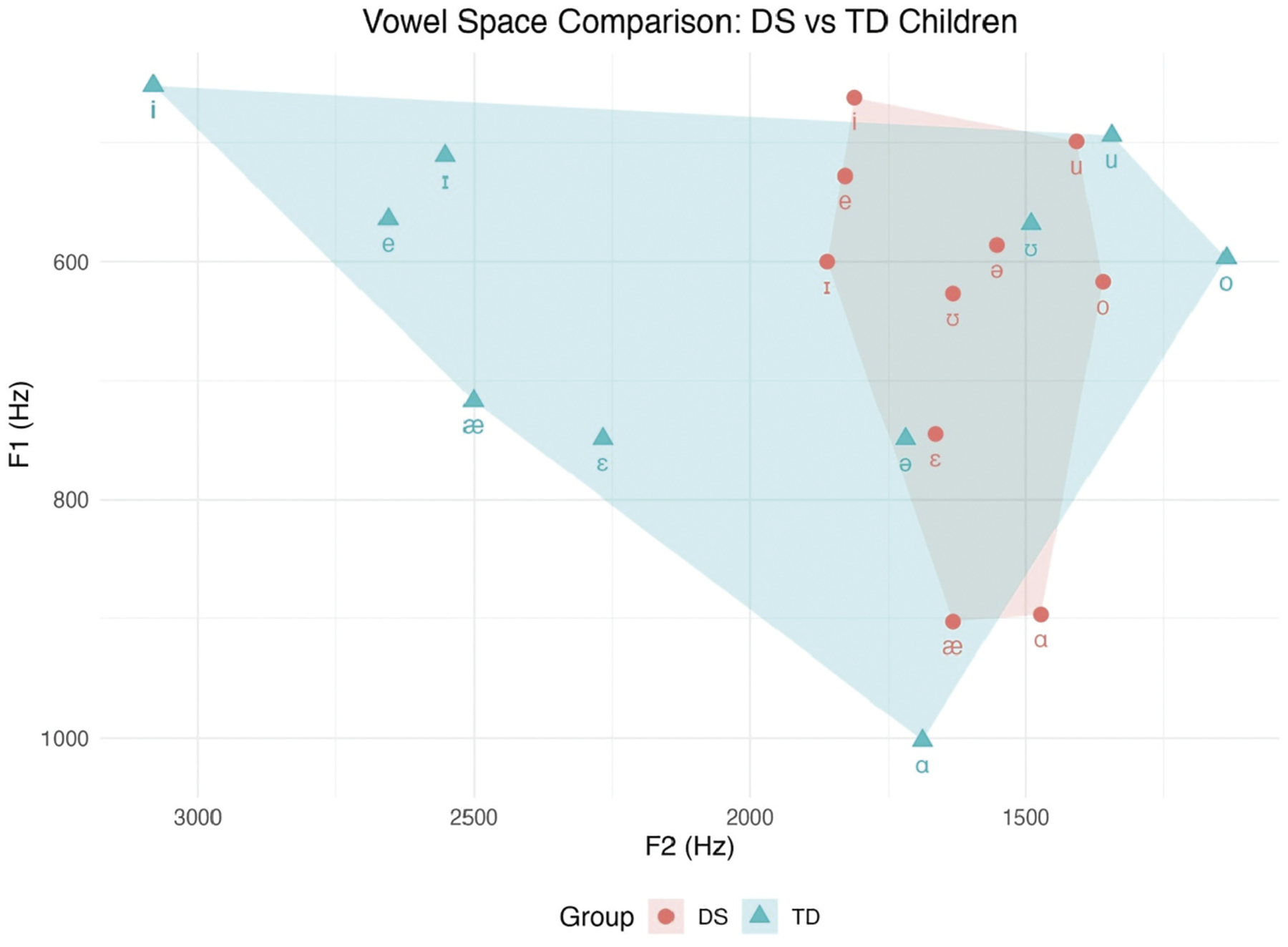
Vowel space comparison for children with DS (baseline session) and TD children aged 10–12 years (from Hillenbrand et al., 1995).

Figure 1 visually reinforces the pattern of vowel centralisation observed in the DS group. Compared to the TD group, vowels in the DS group are clustered more closely together, particularly along the horizontal (F2) axis. This compression is most prominent for front vowels such as /i/, /ɪ/, /e/, and /æ/, which appear substantially less fronted in the DS group. For example, /i/ and /e/ in TD children are clearly separated in the front region of the vowel space, whereas in the DS group, their proximity and lower F2 values suggest reduced tongue advancement and articulatory contrast. A similar pattern is evident among back vowels; while /u/ and /o/ in TD speakers occupy distinct posterior positions, these vowels in the DS group are more centralised and overlapping.

This reduced spatial dispersion of vowel targets highlights a diminished acoustic vowel space in children with DS, which may contribute to lower speech intelligibility. The figure thus complements the tabular data by illustrating how formant compression in DS impacts both vertical (F1) and horizontal (F2) articulatory dimensions in ways that likely hinder perceptual distinction among vowel categories.

Table 3 presents individual-level data for each of the 13 DS participants, showing the change in three vowel space metrics, which are Articulatory-Acoustic Vowel Space Change (ΔAAVS), Quadrilateral Vowel Space Area Change (ΔqVSA), and Convex Hull Vowel Space Area Change (ΔVSAhull), from baseline to post-speech enrichment interventions, alongside their corresponding intelligibility gains as measured by the WCAB AE subtest (WCAB_AE_Gain). Positive values for the vowel space metrics indicate expansion of acoustic vowel space over time, while negative values reflect reductions. WCAB_AE_Gain values represent the difference in GSVs, with higher numbers indicating improved intelligibility. WCAB GSVs are norm referenced gain scores that include weighted raw scores as indices of change (see Kwok et al., 2022). No *a priori* threshold was set for ‘clinical significance’ or ‘meaningful gain’. Instead, GSVs were used to capture the magnitude of gain as an outcome measure for modelling predictors of improvement.

The table reveals considerable variability across participants in both acoustic and perceptual outcomes. Some children, such as DS07 and DS11, showed substantial increases in ΔVSAhull (179,333 and 174,607, respectively) and concurrent gains in WCAB_AE scores (+14 and +3), suggesting a possible association between vowel space expansion and intelligibility improvement. Conversely, other participants, such as DS03 and DS01, exhibited large decreases in all three vowel space metrics alongside declines in WCAB_AE_Gain (−7 and −2, respectively), supporting the idea that reduced articulatory range may be associated with diminished intelligibility.

However, not all children follow this pattern. For example, DS02 showed a large increase in ΔAAVS (+66,802) and ΔVSAhull (+418,538), yet experienced a slight decrease in intelligibility (−1), while DS17 exhibited a strong increase in ΔqVSA (+183,943) but a drop in WCAB_AE_Gain (−3). These discrepancies demonstrate the complexity of speech-motor learning and suggest that other factors, such as vowel accuracy, prosody, or listener variability, may modulate the relationship between vowel space and perceived intelligibility (Borrie et al., 2012; Hustad, 2006; Kent & Kim, 2003). This highlights the need for multifactorial models when interpreting acoustic predictors of functional speech outcomes.

### Vowel space change between assessments

**Figure 2.**
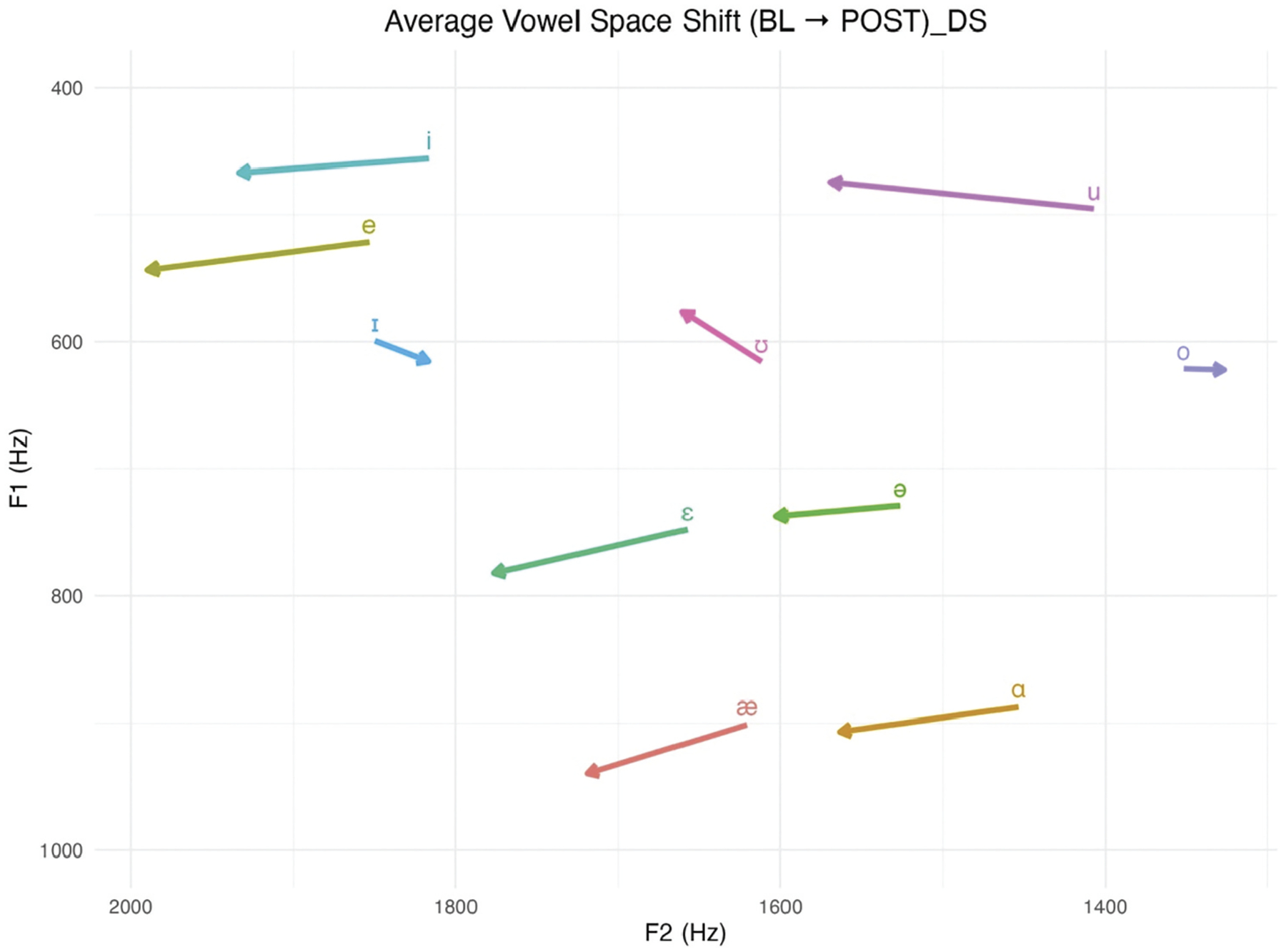
Vowel space shift between baseline and post sessions for DS group.

To visualise how participants’ articulatory patterns changed from pre- to post-speech enrichment interventions, average vowel formant trajectories were plotted across the 10 target vowels. Figure 2 shows the direction and magnitude of formant shifts for each vowel, capturing change in both F1 (vowel height) and F2 (tongue advancement) dimensions.

Figure 2 presents the vowel space change between baseline and post-assessments in the DS group. Arrows indicate average formant movement for each vowel, from the baseline (arrow tail) to the post-speech enrichment interventions (arrowhead). F1 (y-axis) and F2 (x-axis) are both plotted in traditional vowel space orientation, with axes inverted such that higher vowels appear at the top and front vowels at the right.

Across the group, several vowels exhibited systematic directional shifts in formant space, reflecting articulatory reorganisation following speech enrichment interventions. High front vowels such as /i/ and /e/ moved leftward and downward, indicating more fronted and lower tongue positions. Mid-front vowels such as /ɛ/ and low front /æ/ followed a similar trajectory, suggesting increased lowering and advancement of the tongue. Central vowels such as /ə/ and low back /ɑ/ also shifted leftward and downward, pointing to greater fronting and lowering in the vocal tract. The near-high front vowel /ɪ/ and mid back /o/ shifted rightward and downward, consistent with more backed and lower articulations. The high back vowels /u/ and /ʊ/ showed leftward and upward movement, reflecting more fronted and higher productions.

**Figure 3.**
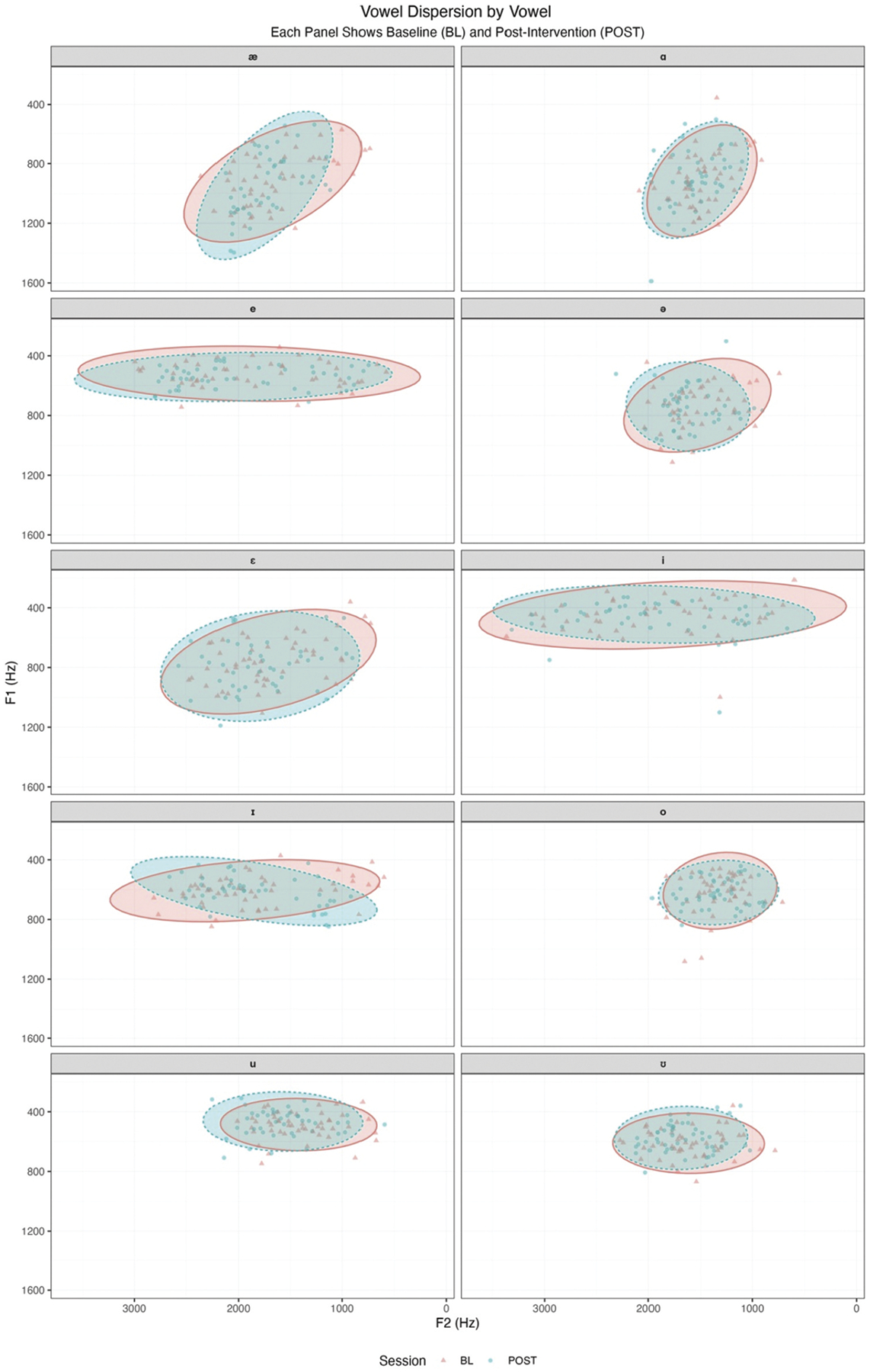
Vowel dispersion by vowel and session.

To complement the average directional shifts reported in vowel space metrics, Figure 3 presents individual vowel productions across sessions, organised by vowel category. Each panel displays baseline and post-intervention productions overlaid with 95% confidence ellipses, providing a clear visualisation of within-vowel dispersion. In many panels, post-intervention productions show reduced scatter and tighter clustering, particularly for high and mid-front vowels, suggesting increased articulatory consistency. These visual patterns align with the AAVS results, reinforcing the interpretation that speech enrichment interventions may support not only expanded vowel space but also more stable and targeted articulatory execution within vowel categories.

### Linear regression results

To assess whether changes in articulatory vowel space predicted gains in speech intelligibility statistically, a multiple linear regression analysis was conducted using change scores from three acoustic metrics: ΔAAVS, ΔqVSA, and ΔVSAhull. The dependent variable was WCAB_AE_Gain, the difference in GSV scores from baseline to post-speech enrichment interventions.

Although some conceptual overlap exists among the vowel space metrics, they capture distinct geometric properties (e.g. centrality, range, and periphery). All three were included in a single model to examine their unique contributions while controlling for potential shared variance. Multicollinearity was assessed and found to be within acceptable limits (all VIFs < 2).

As shown in Table 4, both ΔAAVS (*p* = 0.0275) and ΔVSAhull (*p* = 0.0185) emerged as significant predictors, while ΔqVSA did not (*p* = 0.145). This suggests that changes in articulatory variability and vowel space periphery meaningfully relate to intelligibility gains following speech enrichment interventions.

The directionality of the coefficients offers further insight. The negative coefficient for ΔAAVS indicates that reduced articulatory variability, as reflected in tighter clustering of vowel productions, was associated with greater intelligibility gains. This finding suggests that for children with DS, more stable and consistent articulatory patterns, rather than increased phonetic dispersion, may support clearer speech output.

In contrast, the positive coefficient for ΔVSAhull implies that expanding the overall boundary of vowel space, capturing the articulatory reach across all vowel targets, was positively associated with intelligibility improvement. These results suggest that vowel space dynamics characterised by both greater articulatory reach and reduced internal variability may signal more controlled and intelligible speech production.

These findings highlight the value of using acoustic vowel space metrics that reflect both the breadth and consistency of articulatory gestures, offering clinically interpretable markers of change in speech motor function for children with DS.

## Discussion

### Vowel space expansion following speech enrichment interventions

Children with DS in this study demonstrated measurable changes in articulatory vowel space following naturalistic speech enrichment interventions. Group-level vowel plots showed that vowel movement patterns varied by articulatory class. Several front and central vowels, including /i/, /e/, /ɛ/, /æ/, /ɑ/, and /ə/, shifted towards more fronted and lower tongue positions, while back vowels such as /u/ and /ʊ/ showed movement towards more fronted and higher articulations. Other vowels, including /ɪ/ and /o/, moved in the direction of greater lowering and backing. These diverse directional changes reflect a reorganisation of articulatory patterns rather than a uniform expansion of vowel space. The findings align with previous reports of reduced vowel contrastivity in DS and suggest that targeted speech enrichment interventions may support selective articulatory adjustments (Sorianello, 2015; Vorperian et al., 2023).

Importantly, the extent and nature of vowel space change varied considerably across individuals. While some participants exhibited marked vowel dispersion or boundary expansion, others showed minimal shifts or even reductions in vowel space. This variability likely reflects multiple interacting factors, including baseline articulatory precision, compliance during tasks, and responsiveness to speech enrichment interventions. This highlights the value of individualised tracking over group-level comparisons, especially in populations like DS where speech profiles vary widely. By measuring acoustic change within participants, researchers can capture clinically relevant articulatory shifts that may not yet manifest in global intelligibility ratings or transcription-based scores. Together, these findings highlight the importance of tracking within-participant change and using detailed acoustic measures to capture subtle articulatory shifts that may be overlooked by broader perceptual or transcription-based assessments (Bunton & Leddy, 2011; Whitworth & Bray, 2015).

### Predictive value of vowel space metrics

Among the three acoustic predictors examined, both AAVS and VSAhull significantly predicted intelligibility gains, as measured by WCAB AE Growth Scale Values. However, the directionality of these effects revealed distinct relationships. A positive coefficient for ΔVSAhull indicates that expanding the outer boundary of vowel space, capturing greater articulatory reach across vowel categories, was associated with improved intelligibility. In contrast, the negative coefficient for ΔAAVS suggests that reduced articulatory variability, reflected in more consistent and less dispersed vowel productions, was associated with greater gains in intelligibility.

These patterns suggest that vowel space changes were not uniformly directional but varied by vowel class. The diversity of articulatory shifts likely reflects individual differences in motor flexibility and treatment response. Nonetheless, the overall pattern is consistent with expanded and redistributed vowel space usage following speech enrichment, as shown in Table 3. These acoustic changes correspond with intelligibility gains in several participants and highlight the value of vowel space metrics for monitoring articulatory development in children with DS. Furthermore, this pattern aligns with findings from Carl et al. (2020), who report that vowel cluster variability is significantly associated with intelligibility outcomes in speakers with DS, particularly in adolescent and young adult populations. This reinforces the role of vowel differentiation as a key acoustic correlate of intelligibility across developmental stages in DS.

These findings suggest that intelligibility improvement may be supported not by indiscriminate expansion of acoustic vowel space but by a combination of broader articulatory reach and more stable vowel category formation. That qVSA did not significantly predict intelligibility gain highlights the limitations of traditional quadrilateral metrics, which rely on only four corner vowels and may fail to capture the complexity of articulatory adjustments in this population. In contrast, VSAhull and AAVS incorporate all vowel tokens and provide more comprehensive views of articulatory dynamics. These findings are consistent with research in neurogenic populations and highlight the potential of acoustic vowel space metrics to offer sensitive, interpretable indicators of treatment response in developmental speech contexts (Sandoval et al., 2013; Whitfield & Goberman, 2014).

### Individual variability in intelligibility outcomes

Although group-level results showed a positive association between vowel space expansion and intelligibility improvement, this relationship was not uniform across participants. Some children with considerable vowel space growth did not exhibit parallel gains in WCAB scores. This suggests that vowel space expansion, while important, may not fully account for perceived intelligibility.

Several explanations are possible. First, vowel space metrics index articulatory range but do not directly capture phoneme accuracy, consistency, or timing. Second, WCAB scores reflect whole-word accuracy, which may be influenced by consonant production, prosody, or even listener familiarity. Finally, children may vary in how efficiently they translate articulatory gains into intelligible output depending on cognitive, linguistic, or motor profiles. Research on dysarthria and developmental speech disorders suggests that intelligibility is shaped by multiple interacting features beyond articulatory range. For instance, vowel accuracy (Kent & Kim, 2003), prosodic modulation (Borrie et al., 2012), and listener-related factors such as familiarity or perceptual learning (Hustad, 2006) may strongly influence intelligibility outcomes, even when acoustic patterns suggest improvement. These findings highlight that vowel space expansion does not uniformly translate into intelligibility gains, as perception relies on both acoustic clarity and the listener’s ability to decode speech in context. To fully understand these dynamics, acoustic measures should be integrated with perceptual, phonological, and cognitive assessments within a multidimensional framework, ideally implemented by SLPs to support more tailored and evidence-based intervention planning.

In addition, it is worth considering why a subset of participants demonstrated reduced WCAB scores following the speech enrichment interventions. One possible explanation involves day-to-day fluctuations in attention, behaviour, or task engagement, which are common among children with DS and can impact behavioural test outcomes. Differences in intervention dosage, arising from variations in attendance or the number of completed sessions, may also have contributed to inconsistent gains across individuals. Moreover, WCAB scoring is based on whole-word accuracy and may not always reflect subtle improvements in articulatory precision, particularly if new or emerging errors occurred elsewhere in the word (e.g. in consonant clusters). It is also plausible that some children exhibited positive changes in motor speech patterns that were not immediately perceptible in binary scoring or were offset by challenges in other domains such as memory, executive function, or expressive language planning. These individual-level differences underscore the need for complementary outcome measures that account for both perceptual and cognitive contributors to intelligibility performance.

### Clinical implications

These results carry important implications for speech-language pathology practice. Vowel space metrics, particularly AAVS and VSAhull, may offer clinicians sensitive indicators of articulatory change that precede observable gains in phoneme accuracy or intelligibility ratings. Because these metrics reflect both the range and stability of articulatory gestures, they could complement perceptual assessment tools by revealing progress in clients who may otherwise show minimal change. It is plausible that the BTSR approach used during the speech enrichment interventions contributed to this observed reduction in production variability. By embedding frequent, naturalistic recasts across a broad phonemic inventory, without requiring imitation, the BTSR approach offered consistent, developmentally flexible models of adult-like articulation. This strategy may have supported more stable and refined articulatory gestures over time, particularly in children with DS, who benefit from high-frequency input in a communicative context.

These findings are relevant for treatment planning. For example, approaches such as Speech Intelligibility Treatment (Levy et al., 2021), which emphasises articulatory exaggeration (‘big mouth’ speech), may directly target vowel dispersion and could be adapted for children with DS. Similarly, programs such as Lee Silverman Voice Treatment (LSVT LOUD^®^) (Ramig et al., 2001), originally developed for adults with Parkinson’s disease, have also been applied to children with speech motor challenges, including those with Down syndrome. For example, Boliek et al. (2022) reported positive outcomes following LSVT LOUD in children with DS. These approaches suggest that increased vocal effort may induce changes in vowel articulation and intelligibility (Sapir et al., 2007).

Beyond DS, the use of vowel space metrics may extend to populations with dysarthria, childhood apraxia of speech, or speech production difficulties. In such cases, acoustic markers could provide a non-invasive method of tracking speech motor development or treatment efficacy, especially in naturalistic or low-structure intervention contexts. Future work should explore how vowel space visualisations or real-time feedback might be incorporated into enrichment interventions to scaffold articulatory precision in developmentally diverse groups.

Despite the promise of vowel space metrics, there are practical limitations to their clinical adoption. Measuring formants and calculating vowel space requires specialised software, acoustic analysis training, and time that many clinicians may not have. Perceptual testing remains the gold standard in speech-language pathology due to its efficiency and ecological validity (Hustad, 2006). However, acoustic vowel space measures can reveal subtle articulatory gains that may precede perceptual improvements, offering a more sensitive index of progress for clients whose speech may otherwise appear plateaued. Such early indicators could inform decisions about continuing or adjusting intervention. Future development of clinician-friendly software tools, including automated vowel labelling and visual biofeedback systems that use acoustic parameters (e.g. formant trajectories or vowel space plots), may help bridge the gap between research-grade acoustic analysis and real-world clinical usage.

## Conclusion

This study examined whether changes in vowel space can predict intelligibility gains in children with DS following speech enrichment interventions. Acoustic data from repeated productions of controlled /hVt/ words were used to calculate vowel space at both baseline and post-intervention using three metrics: AAVS, qVSA, and VSAhull. These acoustic measures were then compared to score changes on the WCAB AE subtest, a standardised intelligibility assessment. By modelling both within-participant articulatory changes and between-participant variability, this study evaluated the predictive value of vowel space dynamics in relation to functional speech intelligibility.

Our results revealed that vowel space metrics such as VSAhull and AAVS are sensitive to articulatory changes over time and can meaningfully reflect communicative progress in children with DS. While direct acoustic analysis (e.g. formant plotting) may not be feasible in most clinical settings, these findings support the development of clinician-friendly tools that automate vowel space tracking. By embedding these metrics into software-based assessments, speech-language pathologists could gain access to sensitive indicators of articulatory change that complement perceptual ratings and assist in monitoring subtle treatment effects, especially in populations where intelligibility gains may lag behind motor improvement (Bunton & Leddy, 2011; Vorperian et al., 2023; Whitfield & Goberman, 2014).

This study contributes to a growing body of research suggesting that acoustic phonetic analysis, when translated into accessible formats, can enhance clinical decision-making by revealing early progress in articulatory control, helping guide individualised intervention strategies. However, several limitations exist in the current study. Our sample size was relatively small, which may limit statistical power and the generalisability of findings to the broader DS population. The age distribution of our sample was also skewed, with one participant substantially younger (4 years, 2 months) than the others, who ranged from 8 to 16 years. While this child met all inclusion criteria and completed both baseline and post-treatment assessments, their inclusion may introduce additional developmental variability. Additionally, vowel productions were elicited using a highly structured task involving isolated /hVt/ CVC words, which may not capture the full range of articulatory demands or intelligibility challenges that arise in more complex, connected speech. This is particularly relevant given that children with DS often experience greater difficulty with polysyllabic words and sentence-level constructions. The use of a fixed consonantal frame, while helpful for acoustic consistency, also restricts the phonetic diversity of the task and may limit ecological validity. Moreover, the acoustic analysis was based on just three productions per vowel per participant, which may be sufficient for estimating average vowel space metrics but is less ideal for capturing fine-grained variability, especially when articulatory consistency is a variable of interest. Future studies should consider incorporating larger and more varied speech samples to improve the reliability of variability estimates, as well as more naturalistic speech tasks and more tightly age-stratified sampling to better reflect functional speech performance across the developmental spectrum.

Several avenues remain for future research. First, larger and more diverse samples should be included to improve the generalisability of findings. Second, longitudinal follow-up beyond the immediate post-speech enrichment intervention period would help assess the long-term effects of intervention. Third, further acoustic and perceptual measures should be investigated. For example, examining how vowel space dynamics interact with prosody, coarticulation, and motor planning across different speech contexts may yield deeper insights into intelligibility development in children with DS.

## Figures and Tables

**Table 1.** Demographic characteristics of participants with Down syndrome (DS).

Speaker	Chronological Age (Years & Months)	WCAB Age Equivalent Score (Years & Months)	Sex
DS13	4;2	2;0	male
DS11	8;10	2;0	female
DS01	8;10	2;3	male
DS07	8;9	3;3	female
DS08	8;7	<2;0	female
DS15	11;4	2;8	female
DS10	11;10	<2;0	male
DS18	12;8	2;4	male
DS02	13;2	2;8	female
DS06	13;3	2;4	female
DS03	15;0	2;10	male
DS09	15;0	2;1	female
DS17	16;3	2;8	female

WCAB: Woodcock Camarata Articulation Battery.

**Table 2.** Mean F1 and F2 formant frequencies (Hz) for each phoneme across all speakers in DS (baseline session) and TD (Hillenbrand et al., 1995) groups.

Phoneme	DS		TD	
	F1 (Hz)	F2 (Hz)	F1 (Hz)	F2 (Hz)
i	462	1810	452	3081
ɪ	600	1860	511	2552
ɛ	745	1664	749	2267
e	528	1827	564	2656
æ	903	1632	717	2501
ə	726	1553	586	1719
u	499	1408	494	1345
ʊ	627	1632	568	1490
o	617	1361	597	1137
ɑ	897	1472	1002	1688

F1: first formant; F2: second formant; DS: Down syndrome; TD: typically developing.

**Table 3.** Vowel space change between sessions and intelligibility score gain.

Speaker	ΔAAVS	ΔqVSA	ΔVSAhull	WCAB_AE_GAIN
DS01	−5819.27	−620939.77	−214417.43	−2
DS02	66,801.69	−100302.82	418,538.12	−1
DS03	−27110.35	−101581.55	−304131.37	−7
DS06	−8502.12	−143924.48	−57975.48	3
DS07	103.02	−55755.14	179,333.30	14
DS08	11,885.86	−71054.22	56,110.26	2
DS09	−47592.38	−104539.43	−229482.71	3
DS10	12,218.59	−55715.40	164,950.74	0
DS11	9143.78	81,825.20	174,607.30	3
DS13	−2499.41	−335528.19	−181308.92	4
DS15	−36613.26	11,140.77	−126573.86	2
DS17	18,184.57	183,942.80	36,913.09	−3
DS18	−6629.19	41,777.69	71,942.63	0

ΔAAVS: change in articulatory acoustic vowel space; ΔqVSA: change in quadrilateral vowel space area; ΔVSAhull: change in convex hull vowel space area; WCAB_AE_GAIN: gain in WCAB-Age equivalent score.

**Table 4.** Multiple linear regression predicting WCAB_AE gain.

Predictor	Estimate	Std_Error	t_value	p_value
(Intercept)	−0.027	1.368	−0.020	0.9847
ΔAAVS	−0.00022	0.000084	−2.628	0.0275*
ΔqVSA	−0.00001	0.000007	−1.596	0.145
ΔVSAhull	0.00004	0.000013	2.870	0.0185*

Asterisks indicate significance levels (*: *p* < 0.05; **: *p* < 0.01; ***: *p* < 0.001).

## Data Availability

The data that support the findings of this study are available from the corresponding author, DD, upon reasonable request.

## Footnotes

1 We use /ə/ to represent the STRUT vowel phoneme (as in ‘hut’) in our broad phonemic transcription. While /ʌ/ is the conventional symbol for this vowel in stressed syllables, we chose /ə/ to reflect the consistent phonetic realisation observed in our participant group, specifically, a mid-central unrounded vowel. We acknowledge that /ə/ is typically reserved for reduced vowels in unstressed contexts, and that it is not the standard phonemic representation of the STRUT vowel. However, because our monosyllabic stimuli lacked stress contrast, and the phonetic quality of this vowel was consistently centralised, /ə/ was used throughout for consistency in transcription and vowel space plotting.
